# Causation of increased prostate cancer in young men

**DOI:** 10.18632/oncoscience.527

**Published:** 2021-03-20

**Authors:** Archie Bleyer, Filippo Spreafico, Ronald Barr

**Affiliations:** ^1^Department of Radiation Medicine, Knight Cancer Institute and Oregon Health & Science University, Portland, Oregon, USA; ^2^McGovern Medical School, University of Texas, Houston, Texas, USA; ^3^Department of Medical Oncology and Hematology, Pediatric Oncology Unit, Fondazione IRCCS Istituto Nazionale dei Tumori, Milan, Italy; ^4^Departments of Medicine, Pediatrics, and Pathology, McMaster University and McMaster Children’s Hospital, Hamilton, Ontario, Canada

**Keywords:** prostate cancer incidence, young men, cause, overdiagnosis

In our original report [1] we described how, between 1992 and 2015, prostate cancer incidence increased in all men <50 years of age and decreased in those >50 years of age. We also reported that many other countries had evidence for increasing prostate cancer in young men. We speculated that the possible causes included improved recognition (prior under-diagnosis), overdiagnosis, PSA screening, human papilloma virus infection, racial/ethnic/familial factors, environmental carcinogens, obesity, sedentary behavior, and societal factors. Since then and with an additional year of data and more detailed analyses, we now suspect that overdiagnosis and PSA screening contributed more than the other possibilities, even though PSA screening was not commonly considered before age 45 or 50 in the general population.

**Figure 1:**
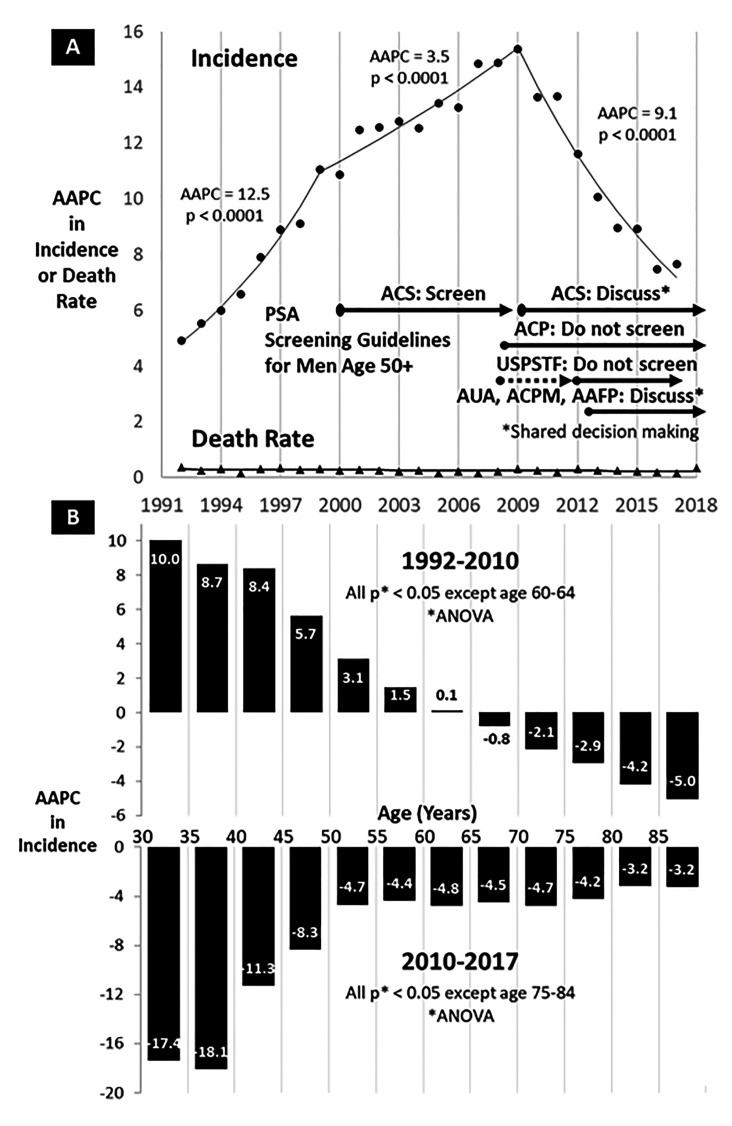
Joinpoint/Average Annual Percent Change (AAPC) in Pancreas Cancer in Men, SEER13. **(A)**. Annual Incidence, 1992-2017, and Annual Death Rate, 1992-2018, Age 30-49 and American Cancer Society (ACS), American College of Physicians (ACP) as in (ACP). U.S. Preventative Services Task Force (USPSTF), American Urological Association (AUA), American College of Preventive Medicine (ACPM) and American Academy of Family Physicians (AAFP) PSA Screening Guidelines. (B). Annual Incidence, 1992-2010 and 2000-2017, by 5-Year Age Intervals. Data source: SEER*Stat Database [2].

As shown in Figure 1A, prostate cancer incidence in men 30-49 years of age increased more than 3-fold from 1992 to 2010, at an average annual percent change (AAPC) of 12.5 during 1992-1999 (p<0.0001) and 3.5 during 1999-2010 (p<0.0001) [2]. Yet there was no evidence for change in prostate cancer mortality in the age group during the incidence increase. Indeed, joinpoint regression revealed no changes in the death rate over the entire interval of 1992-2018 (Figure 1A). This incidence-mortality comparison is classic for overdiagnosis, with no evidence that the dramatic increase in incidence eventuated in a death increase despite the much greater number of cases. If so, the additional “over-diagnosed” cases would not have affected the over-diagnosed men anytime in the rest of their lives.

In 2010 the incidence trends reversed, not only in young men but also those over 50 years of age. In 30-49 year-olds, the decrease was dramatic, by 50% in 7 years at an AAPC of 9.1 during 2010-2017 which, according to joinpoint analysis, was an exponential decline (Figure 1A). The decrease occurred when multiple national organizations changed their recommendations for PSA screening of men at average risk of prostate cancer. The American Cancer Society [3], American American Academy of Family Physicians [4], American College of Preventative Medicine [5] and American Urological Association [6] recommended shared decision making (discussion between patient and provider) instead of routine screening and the American College of Physicians [7] and U.S. National Preventative Task Force on Prostate Screening [8-10] advised that routine screening be discontinued altogether (Figure 1A). These recommendations applied to men over 50 years of age who previously had been recommended to be routinely screened. So, how could this affect younger men?

As we described, the U.S. CDC reported that, during 2000-2015 in the U.S., 2% of men aged 30-39 and 5%-6% of men aged 40-49 with health insurance were “screened” with PSA, “contrary to all existing practice guidelines” and “despite all medical organizations recommending against PSA screening of men younger than 50 and older than 69” [11]. “Screening” is not an accurate description of this small percentage since they likely had PSA levels obtained because of a family history of prostate cancer or were otherwise clinically suspicious and were not part of population screening. The CDC investigators found that there was a significant temporal trend of PSA testing in men aged 30-39 years aged 40-49 years (p = 0.005), while in all older age groups the rate of PSA testing declined proportional to increasing age. The decline in older men is likely due to a U.S. Preventative Services Task Force (USPSTF) recommendation in 2008 against screening men over 75 years of age [8, 9], a recommendation that was endorsed by the American College of Preventive Medicine [5] and the American Academy of Family Physicians [4]. If this practice recommendation influenced PSA testing in younger men, it should have reduced testing in them too since it was never recommended for them and the guidelines rendered this practice even more questionable. Also, effective screening increases the proportion of patients with early disease, which is the opposite of the pattern by age we observed in young men [1]. On the other hand nearly all the increase was of earlier stage disease and not of late stage with distant metastases, which is consistent with a PSA screening effect.

An observation we did not emphasize in our original report is how directly and inversely proportional by age the increase and subsequent decrease occurred in men under 55 years of age (Figure 1B). In men 30-39 years of age the AAPC was an increase of 8.7-10.0 before 2010 and a decrease of 17.4-18.1 afterwards (Figure 1B). That the trend effects were greatest in the youngest men may seem inconsistent with national PSA screening recommendations in men older than 50. That they were reciprocally proportional to age, however, supports the overdiagnosis explanation. It may also be due, however, to greater concern in the youngest men since men considerably older than 40 were having their routine screening decreased and in men older than 65-70 years discontinued. Some of the increase in the youngest men was likely due to PSA testing in men at higher prostate cancer risk, as genomic information became more available to identify higher-risk men. This explanation is supported by our observation that the youngest men presented with a worse stage and had worse survival [1]. Also, other countries have had similar prostate cancer increases in their young men1 that may not have had national PSA screening programs to the extent of the U.S. and, if so, other factors explain their increase such as racial/ethnic/familial factors, HPV infection, carcinogens, obesity, and sedentary behavior. Overall, however, most of the increase in American men appears to have been due to overdiagnosis.
